# Benzimidazole inhibitors of protein kinase CK2 potently inhibit the activity of atypical protein kinase Rio1

**DOI:** 10.1007/s11010-016-2892-x

**Published:** 2016-12-01

**Authors:** Konrad Kubiński, Maciej Masłyk, Andrzej Orzeszko

**Affiliations:** 1 Department of Molecular Biology, Institute of Biotechnology, The John Paul II Catholic University of Lublin, ul. Konstantynów 1i, 20-708 Lublin, Poland; 2 Institute of Chemistry, Warsaw Life Sciences University, ul. Nowoursynowska 159c, 02-787 Warsaw, Poland

**Keywords:** Protein kinases, Small-molecule inhibitors, Competitive inhibition, Toyocamycin, TIBI

## Abstract

Benzimidazole derivatives of 5,6-dichlorobenzimidazole 1-β-d-ribofuranoside (DRB) comprise the important class of protein kinase CK2 inhibitors. Depending on the structure, benzimidazoles inhibit CK2 with different selectivity and potency. Besides CK2, the compounds can inhibit, with similar activity, other classical eukaryotic protein kinases (e.g. PIM, DYRK, and PKD). The present results show that a majority of the most common CK2 inhibitors can affect the atypical kinase Rio1 in a nanomolar range. Kinetic data confirmed the mode of action of benzimidazoles as typical ATP-competitive inhibitors. In contrast to toyocamycin—the first discovered small-molecule inhibitor of Rio1—the most potent representative of benzimidazoles TIBI (IC_50_ = 0.09 µM, *K*_*i*_ = 0.05 µM) does not influence the oligomeric state of the Rio1 kinase. Docking studies revealed that TIBI can occupy the ATP-binding site of Rio1 in a manner similar to toyocamycin, and enhances the thermostability of the enzyme.

## Introduction

Protein kinases play important roles in key cellular processes, including the cell cycle, metabolism, and cell death [1, 2]. The protein kinase superfamily, with 518 members, comprises one of the largest protein superfamilies identified in the human genome [3]. In addition to their key roles in cell physiology, about half of protein kinases are linked to pathological states, including cancer [4, 5]. This fact makes kinases attractive targets for therapeutic intervention.

One such kinase therapeutic target is probably the most pleiotropic protein kinase, CK2. It phosphorylates more than 300 protein substrates and thus remarkably increases the level of total protein phosphorylation in the cell [6, 7]. Interestingly, the enzyme is constitutively active, and the mode of its on/off mechanism is unknown [7, 8]. The plethora of cellular partners makes protein kinase CK2 engaged in a majority of cellular processes, both physiological, such as the cell cycle, and pathological ones, e.g. viral infection or cancer development [9]. Elevated expression and activity levels of CK2 have been reported in many human pathologies, including cancer cells (reviewed in [10]).

Benzimidazole derivatives represent the biggest family of ATP-competitive inhibitors of protein kinase CK2, with a scaffold derived from the 5,6-dichloro-1-(β-d-ribofuranosyl)benzimidazole (DRB) molecule [11]. Starting from this molecule, the structure of the inhibitors has been optimized to better fit in the ATP-binding pocket [12]. In this respect, the most successful commercially available benzimidazole CK2 inhibitors are TBB, TBI, and DMAT [13]. Benzimidazole derivatives effectively inhibit both native and recombinant CK2 activity in vitro and in cell culture showing proapoptotic properties [14, 15]. Although benzimidazole derivatives are potent inhibitors of CK2, TBB, TBI, and even the most selective DMAT show activity towards several other kinases, such as PIM, DYRK, or PKD [13].

The protein kinase superfamily includes 40 members that are classified as atypical protein kinases. One such enzyme is the Rio1 kinase, the founding member of the RIO family that also comprises Rio2, Rio3, and RioB [16, 17]. Rio1 is involved in ribosome biogenesis, cell cycle progression, and chromosome maintenance [18–20]. Studies on yeast revealed that Rio1 is a non-ribosomal factor that is essential for the processing of 20 S pre-rRNA to 18 S rRNA, a component of the 40 S subunit [21, 22]. Little is known about the physiological role of Rio1-mediated phosphorylation, cellular protein substrates of the enzyme, and the physiological role of Rio1 autophosphorylation. Although the possible role of Rio1 in pathogenesis is not well established in comparison to many other kinases, it was reported that the enzyme is upregulated in colon cancer, and there is a direct link between deregulation of ribosome biogenesis and tumour development [23]. In other studies, Read and coworkers showed that the kinases Rio1 and Rio2 are overexpressed in glioblastoma cells in an Akt-dependent manner and promote tumorigenesis [24].

Toyocamycin was the first identified small-molecule inhibitor of Rio1, showing mixed inhibition. This mode of action of toyocamycin results from its dual activity towards the Rio1 kinase. On the one hand, toyocamycin acts as an ATP-competitive inhibitor, and on the other hand, it stabilizes the less catalytically active oligomeric isoform of the Rio1 kinase [25]. Recently, several pyridine caffeic acid benzyl amides (CABA) have been identified as a novel molecular probes of the Rio1 following the ATP-competitive inhibition mode [26].

Here we present a series of benzimidazole CK2 inhibitors as novel synthetic small-molecule inhibitors of the Rio1 kinase. We analysed the kinetic data of Rio1 autophosphorylation in the presence of the most active compound TIBI, with respect to the inhibition model. In order to verify the influence of TIBI on the thermostability of human Rio1, we determined the melting temperature of the enzyme in the presence and absence of the inhibitor. We also employed size-exclusion chromatography to validate whether TIBI is able to affect the oligomeric state of Rio1. Moreover, computational studies were used in order to calculate possible kinase–ligand complexes.

## Materials and methods

### Reagents and benzimidazole CK2 inhibitors

All reagents were purchased from Sigma-Aldrich. 4,5,6,7-Tetrabromo-2-azabenzimidazole, (TBB), 2-dimethylamino-4,5,6,7-tetrabromobenzimidazole (DMAT), and toyocamycin were purchased from Sigma-Aldrich. CX-4945 (Silmitasertib) was purchased from APEXBIO. All the other benzimidazoles studied were synthesized in our laboratory previously and have already been described as follows:

- *TIBI*, (4,5,6,7-tetraiodobenzimidazole) [27].
- *K92*, (4,5,6,7-tetraiodo-2-methylbenzimidazole), [27].
- *TI-2Am*, (4,5,6,7-tetraiodobenzimidazole-2-amine-ethylamine), [28].
- *TidiMe*, (2-dimethylamino-1-metyl-4,5,6,7-tetraiodobenzimidazole), [28].
- *K95*, (4,6-dibromo-5,7-diiodobenzimidazole), [27].
- *TBI* (4,5,6,7-tetrabromobenzimidazole), [29].
- *TCI*, (4,5,6,7-tetrachlorobenzimidazole), [30].
- *TDBB* (3-methyl-4-[4,5,6,7-tetrabromo-2-(dimethylamino)benzimidazole-1-yl]butanoic acid, [31].
- *TBSB*, (3-(4,5,6,7-tetrabromobenzimidazole-2-yl sulfanyl)butanoic acid), [31].
- *1Me-DMAT*, (4,5,6,7-tetrabromo-*N*,*N*,1-trimethylbenzimidazole-2-amine), [12].
- *TBTS*, [(4,5,6,7-tetrabromo-*N*,*N*,1-trimethylbenzimidazole-2-yl)sulfanyl]acetic acid, [31].

### Kinase activity assays

Recombinant human protein kinase GST-CK2α was produced in bacteria and purified as described elsewhere [32]. The plasmid carrying the gene encoding human protein kinase Rio1 (residues 143–494) was kindly gifted by Professor Nicole LaRonde—LeBlanc (University of Maryland, USA). Human recombinant kinase Rio1 containing residues 134–494 was expressed and purified as previously reported with a few modifications [33].

In the case of CK2, the phosphorylation reactions were conducted at 37 °C for 5 min in 50 μl samples each containing 1 pmol of human recombinant CK2α, 10 µg of ribosomal P2B protein as a substrate, and an appropriate concentration of the tested benzimidazole (0.01–50 µM). The reaction buffer contained 20 µM [γ-^32^P]ATP (specific radioactivity 300–1000 cpm/pmol), 15 mM Mg^2+^, 20 mM Tris–HCl pH 7.5, and 6 mM 2-mercaptoethanol.

In order to determine the autophosphorylation of human Rio1, the enzyme was incubated at 37 °C for 15 min in 50 μl samples each containing 1 pmol of human recombinant Rio1 and an appropriate concentration of tested compound (0.01–50 µM). The reaction buffer contained 0.5 µM ATP, [γ-^32^P]ATP (specific radioactivity 300–1000 cpm/pmol), 50 mM NaCl, 10 mM Mg^2+^, 50 mM Tris–HCl pH 7.5, and 6 mM 2-mercaptoethanol.

In order to determine the *K*_m_ of Rio1 for ATP, Rio1 was incubated for 5 min in the presence of increasing concentrations of ATP (0.001–1 µM) in a reaction buffer containing 50 mM NaCl, 10 mM Mg^2+^, 50 mM Tris–HCl pH 7.5, 6 mM 2-mercaptoethanol, and 1 µCi of [γ-^32^P]ATP. Additionally, the same protocol was followed with the addition of TIBI to the reaction mixture at a concentration of 0.01, 0.05, and 0.1 µM. The use of such low concentrations of ATP in reaction mixtures was optimal for Rio1, and resulted from the inhibition of Rio1 activity at ATP concentration greater than 1 µM [25, 26].

The phosphorylation was terminated by addition of SDS/PAGE sample buffer, and proteins were resolved by electrophoresis, followed by Coomassie Brilliant Blue staining. The gels were dried and ^32^P-labelled bands of P2B and Rio1 were excised from the gel, and radioactivity was determined in a scintillation counter (MicroBeta, Perkin Elmer) by Cerenkov counting.

Kinetic data were obtained using enzyme preparations from three purification batches.

All experiments were performed in triplicate.

IC_50_, *K*_*i*_, *K*_m_, and *V*_max_ values were calculated using GraphPad Prism (version 4.0) software.

### Thermofluor assay

The procedure was performed according to the protocol described elsewhere with a few modifications [25]. 10-µl reactions containing 50 mM NaCl, 10 mM Mg^2+^, 50 mM Tris–HCl pH 7.5, 1 mg/ml hRio1 or hCK2α, and 100 µM TIBI or 5% DMSO (control) were incubated at 4 °C for 30 min. 10 µl of the SYPRO Orange dye was then added to the reactions. The samples were heated from 4 to 98 °C at a rate of 0.2° per second using a thermal cycler (CFX 1000, Bio-Rad). The changes in fluorescence intensity were measured as the temperature increased. Melting temperatures (*T*_m_) of hRio1 and CK2 in the presence and absence of TIBI were calculated using Bio-Rad software. The experiment was performed in duplicate.

### Size-exclusion chromatography

The glass column (200/10 mm) filled with the Sephadex G-200 medium (GE Healthcare) was equilibrated with a buffer containing 10 mM Tris, pH 7.5, 150 mM NaCl, and 6 mM β-mercaptoethanol. In order to calibrate the column, four proteins, namely β-amylase (200 kDa), alcohol dehydrogenase (150 kDa), bovine serum albumin (67 kDa), and ovalbumin (43 kDa), were separately loaded onto the column. 100-µl samples containing 2 mg/ml hRio1 diluted in the equilibration buffer in the presence or absence (control) of 100 µM TIBI were then loaded onto the column. The chromatography was performed at a flow rate 0.2 ml/min using the FPLC system AKTA Purifier 10 (GE Healthcare), and runs were monitored by OD_280_.

### Computational studies

For molecular docking, we selected the X-ray structure of CK2 in complex with ANP (PDB ID: 3NSZ) and Rio1 in complex with ADP (PDB ID: 4OTP) as target proteins for the initial docking studies. All Mg^2+^ ions were removed in the 3NSZ and 4OTP experimental structures as well as all sulphates, co-solvents, water molecules, and original ligands. The structures were then minimized using YASARA Energy Minimization Server [34]. Autodock Tools v1.5.6 (The Scripps Research Institute) was used for charging the proteins as well as ligands. Docking calculations were performed with Autodock Vina v1.1.2 (The Scripps Research Institute) under default conditions [35]. During the docking calculations, all the protein residues were fixed and only the inhibitor atoms were allowed to move. Visualization of the binding site complexed with the docked ligand was performed by Maestro Suite and PyMOL v1.2 (Schrödinger) software.

## Results and discussion

### Benzimidazole inhibitors of protein kinase CK2 suppress the autophosphorylation of an atypical human kinase Rio1

Halogenated benzimidazoles are widely reported as CK2 inhibitors that (i) downregulate both native and recombinant protein kinases CK2 from various sources [36], (ii) can discriminate between different isoforms of the enzyme in vitro [31, 37], (iii) induce apoptosis in many cancer cell lines [38, 39], and (iv) can inhibit other classical eukaryotic protein kinases with different potency [13]. Our results show that the atypical protein kinase (the most stable form of Rio1 containing residues 134–494) is susceptible to benzimidazole inhibitors of CK2 (Table 1). With the exception of TDBB, TCI, and TBB, the tested compounds suppress the autophosphorylation of the Rio1 kinase in a nanomolar range. Although TBB inhibits CK2 ca. 10 times more strongly (IC_50_ = 0.19 µM, *K*_*i*_ = 0.081 µM) than Rio1 (IC_50_ = 1.74 µM, *K*_*i*_ = 0.983 µM), the atypical kinase belongs, together with Pim1 and Pim3, to the most sensitive kinases to TBB action [13]. TCI displays the least efficacy towards the two tested kinases, which is consistent with previous reports showing that chloride derivatives of benzimidazoles are not as efficient CK2 inhibitors as other halogenated derivatives [36]. The majority of the other nanomolar inhibitors behave more similarly against CK2 and Rio1 with a slight advantage of the former one. The inhibition of CK2 by TI-2Am was ca. sixfold stronger than that of Rio1. In turn, K92 induced fourfold stronger inhibition of CK2 than that of Rio1, and in the case of 1Me-DMAT and TBSB, the inhibition of CK2 was twofold stronger than that of Rio1. The other compounds showed almost the same efficacy towards both kinases, and TIBI with IC_50_ = 0.09 µM and *K*_*i*_ = 0.05 µM appeared to be the most potent inhibitor of Rio1. We observed that higher potency towards the atypical kinase was shown by the iodine derivatives of benzimidazole (IC_50_ between 0.09 and 0.24 µM, *K*_*i*_ values between 0.05 and 0.135 µM) than by the bromide derivatives (IC_50_ between 0.19 and 2.17 µM, *K*_*i*_ values between 0.1 and 1.226 µM), with no significant changes caused by the addition of methyl (K92, TIdiMe) or amino groups (TI-2Am). One of the most potent inhibitors of Rio1 is the commercially available DMAT (IC_50_ = 0.19 µM, *K*_*i*_ = 0.1 µM), which appeared to be not as selective towards CK2 (IC_50_ = 0.19 µM, *K*_*i*_ = 0.081 µM) as TBB, which is consistent with data presented previously by Pagano and coworkers [13]. Selected benzimidazoles appeared to be more potent (up to ca. 20-fold) toward Rio1 than the first identified inhibitor of Rio1, toyocamycin (IC_50_ = 3.66 µM, *K*_*i*_ = 2.068 µM), or 20- to 120-fold stronger in comparison to the recently developed Rio1 probes - pyridine caffeic acid benzyl amides (CABA) (IC_50_ from 3.8 to 23.7 µM). Thus, derivatives of halogenated benzimidazoles may constitute a novel class of inhibitors of protein kinase Rio1 [25, 26].

**Table 1:** IC_50_ and K_i_ determinations for benzimidazole derivatives against protein kinases Rio1 and CK2

Inhibitor	Structure	IC_50_ (µM) ± SD		K_i_ (µM) ± SD	
		Rio1	CK2	Rio1	CK2
TIBI		0.09 ± 0.005	0.083 ± 0.003	0.05 ± 0.002	0.035 ± 0.001
K92		0.19 ± 0.011	0.066 ± 0.003	0.1 ± 0.004	0.025 ± 0.001
DMAT		0.19 ± 0.009	0.19 ± 0.008	0.1 ± 0,003	0.081 ± 0.003
TI-2Am		0.23 ± 0.01	0.050 ± 0.002	0.13 ± 0.005	0.021 ± 0.001
TIdiMe		0.24 ± 0.008	0.20 ± 0.006	0.135 ± 0.005	0.085 ± 0.003
K95		0.29 ± 0.009	0.30 ± 0.009	0.164 ± 0.007	0.128 ± 0.005
TBI		0.33 ± 0.007	0.44 ± 0.01	0.186 ± 0.009	0.188 ± 0.007
1Me-DMAT		0.61 ± 0.02	0.39 ± 0.012	0,344 ± 0.011	0.167 ± 0,009
TBSB		0.65 ± 0.03	0.33 ± 0.013	0.367 ± 0.012	0.141 ± 0.005
TBTS		0.69 ± 0.,2	0.60 ± 0.03	0.39 ± 0.011	0.257 ± 0.009
TBB		1.74 ± 0.05	0.19 ± 0,007	0.983 ± 0.03	0.081 ± 0.002
TCI		1.9 ± 0.07	9.7 ± 0.3	1.07 ± 0.04	4.15 ± 0.1
TDBB		2.17 ± 0.06	1.51 ± 0.04	1.226 ± 0.03	0.647 ± 0.014
Reference compounds					
Toyocamycin		3.66 ± 0.09	54.78 ± 1.9	2.068 ± 0.07	23.47 ± 0.8
CX-4945		0.37 ± 0.011	0.005 ± 0.0001	0.209 ± 0.008	0.002 ± 0.00007

In order to make complete representation of the link between Rio1 and CK2, two reference compounds toyocamycin and CX-4945 were also used [25, 40]. Protein kinase CK2 was quite refractory (IC_50_ = 55 µM, *K*_*i*_ = 23.48 µM) to toyocamycin, the first small-molecule inhibitor of kinase Rio1, while the atypical kinase showed sensitivity (IC_50_ = 0.37 µM, *K*_*i*_ = 0.209 µM) towards CX-4945—the most potent inhibitor of the protein kinase CK2, currently being in clinical trials. Although the nanomolar range of the IC_50_ value may suggest intriguing potency of CX-4945 towards Rio1, the compound is a much more potent inhibitor of several other kinases, with CK2 on the top [40–42].

### TIBI as an ATP-competitive inhibitor of Rio1, and its influence on oligomerization of the enzyme

**Fig. 1:**
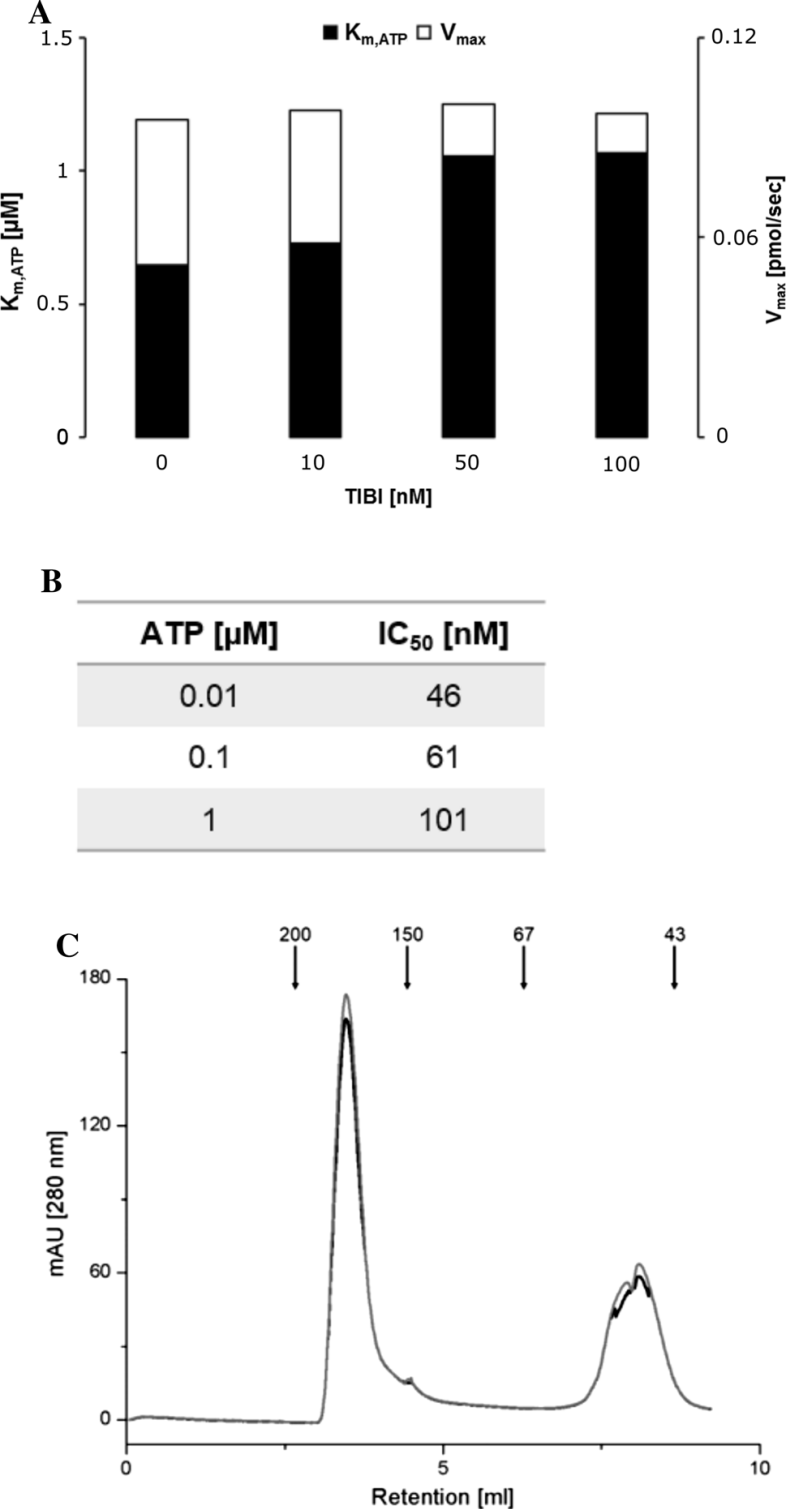
Steady-state analyses of Rio1. **a** *K*_m_ for ATP and *V*_max_ of Rio1 at the increasing concentration of TIBI. **b** IC_50_ determinations for TIBI against Rio1 at different ATP concentrations. **c** Size-exclusion chromatography plots for Rio1 with (*black line*) and without (*grey line*) TIBI. The positions of molecular weight standards (kDa) are indicated with *arrows*

In order to determine whether the well-known ATP-competitive inhibitors of protein kinase CK2 act in the same mode towards Rio1, we determined the steady-state kinetic parameters for the kinase (*K*_m_ for ATP and *V*_max_ of Rio1) in the presence and absence of TIBI. To calculate these parameters, the assay of Rio1 autophosphorylation reaction was used and the level of phosphate (P-32) incorporated into Rio1 was measured. We observed that *K*_m_ for ATP began to rise with increasing TIBI concentrations, starting with 0.65 µM (at 0 µM TIBI - control) to 1.07 µM at 100 µM TIBI (Fig. 1a). Simultaneously, we did not notice any shift in the *V*_max_ value remaining at the same level of 0.09 pmol/sec with the increasing TIBI concentration. To confirm the ATP-competitive nature of the TIBI action, we measured the IC_50_ values of TIBI for three different ATP concentrations. As expected, the parameter showed a dependence of the ATP content; IC_50_ for TIBI increased with the increasing ATP concentrations as follows: 46, 61, and 101 nM at 0.01, 0.1, and 1 µM of ATP, respectively (Fig. 1b). Such low ATP concentrations used in this experiment result from the sensitivity of the kinase Rio1 to ATP concentration higher than 1 µM [25, 26]. Since the results obtained correspond to the linear relationship between IC_50_ and ATP described in the Cheng and Prusoff equation, TIBI and most probably the other tested benzimidazoles act as strict ATP-competitive inhibitors of the atypical kinase Rio1 [43].

Based on the data from the size-exclusion chromatography, we observed that human Rio1 (aa 134–494) can exist in two isoforms, i.e. a monomer (49 kDa) and a dominant tetramer (185 kDa) (Fig. 1c). Similar observation by Kiburu and LaRonde revealed that unphosphorylated human and archaeal Rio1 forms higher order oligomers, while phosphorylation promotes monomerization [25]. In contrast to the recently evaluated inhibitors of the Rio1 kinase (toyocamycin, CABA), TIBI seems not to function as an allosteric inhibitor of Rio1 promoting less active oligomeric states of the kinase [26]. In the case of hRio1 treated with TIBI, we did not observe any changes in the monomer–tetramer equilibrium, compared with the intact enzyme. This observation is consistent with the kinetic data indicating an ATP-competitive mode of TIBI and thus benzimidazole action in Rio1 inhibition.

### TIBI can occupy the ATP-binding site and enhances the thermostability of protein kinase Rio1

**Fig. 2:**
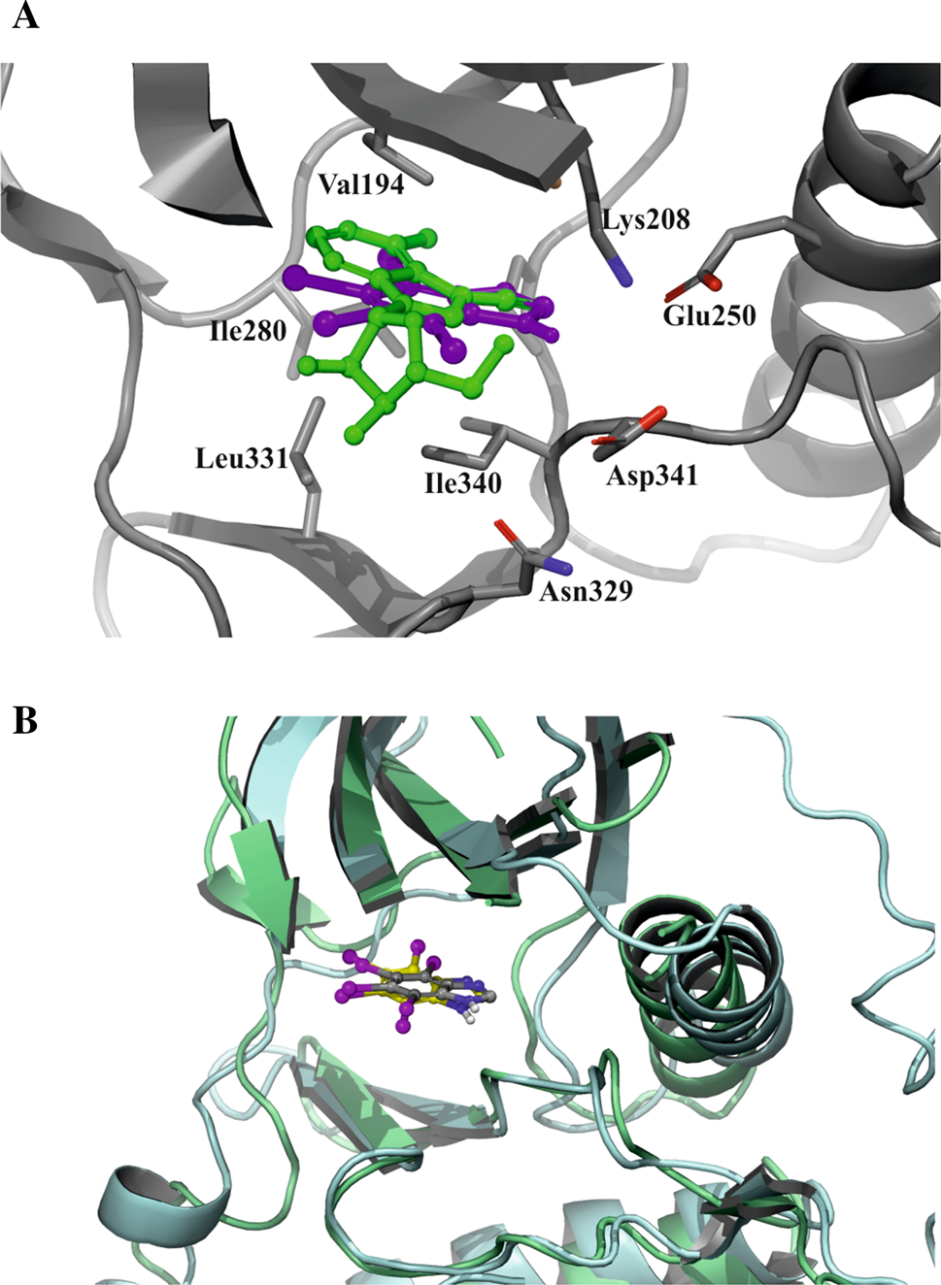
Docked binding mode obtained with Autodock Vina for **a** TIBI (*purple*) and toyocamycin (*green*) in Rio1, **b** TIBI in Rio1 (*yellow* carbons and *green* cartoon) and CK2 (*grey* carbons and aquamarine cartoon). (Color figure online)

In order to rationalize the biological results discussed above, docking studies were performed with the TIBI compound and toyocamycin. The docking calculations of TIBI and Rio1 rendered a common pose for the benzotriazole moiety within the ATP-binding site in kinase CK2 described in resolved crystal structures (Fig. 2b) [30]. The main core of the inhibitor is lodged deep in the binding pocket establishing Van der Waals interactions with the side chains of Leu331, Ile340, Ile186, Val194, Ile280, Pro286, Pro265, Ala206, and Met277 within the adenine-binding site. In turn, the diazole ring is oriented towards the hydrophilic area composed of side chains of Lys208, Glu250, and Asp341. Similar results were obtained for toyocamycin. The main adenosine ring occupies the same space as the benzotriazole ring of TIBI, while the charged sugar ring faces the bulk solvent at the entrance to the ATP-binding pocket, where the side chains of Ser287, Asp341, and Ans329 are located (Fig. 2a).

**Fig. 3:**
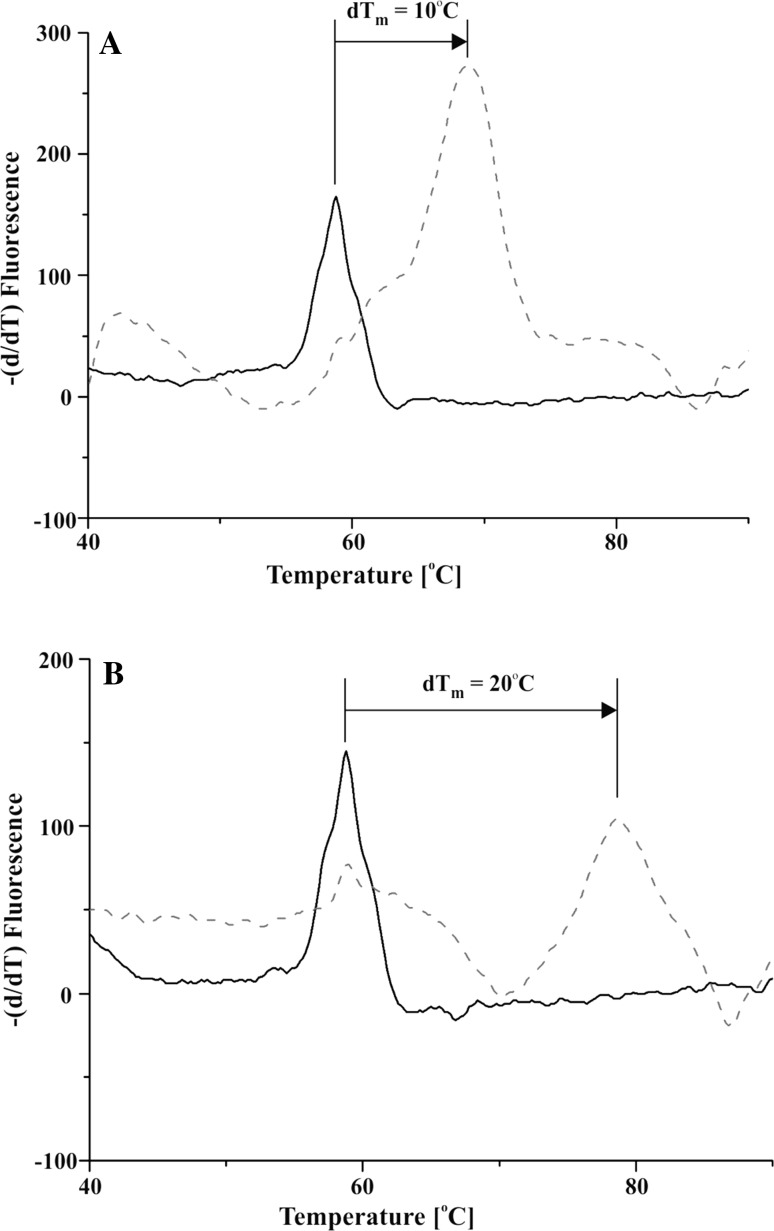
Melt curve derivative plots for **a** Rio1 and **b** CK2. Protein kinases were analysed using thermal shift assays in the absence (*solid lines*) and presence (*dashed lines*) of TIBI. The shifts in *T*_m_ are indicated with *black arrows*

The thermal shift assay revealed that TIBI—the novel benzimidazole inhibitor of human Rio1—significantly enhanced the thermostability of the kinase (Fig. 3a). We observed a shift of 10 °C in the melting temperature (*T*_m_) of bound Rio1 (68.8 °C) in comparison to the unbound enzyme (58.8 °C). The results obtained correspond to data presented by Kiburu and LaRonde, who reported a shift of 12.1 °C in *T*_m_ in the case of the human Rio1 bound to toyocamycin [25]. Simultaneously, we compared the Rio1 with CK2 with respect to the TIBI-mediated changes in the thermostability of proteins. On the one hand, as it is described above, TIBI shows similar potency towards CK2α and Rio1, which reflects the IC_50_ values, i.e. 0.083 and 0.09 µM, respectively. On the other hand, a shift of 20 °C in the melting temperature (*T*_m_) of TIBI-bound CK2α (78.8 °C) in comparison to the unbound enzyme (58.8 °C) was observed (Fig. 3b). Thus, TIBI when bound to CK2α stabilizes structure of the enzyme to a greater degree than in the case of Rio1.

### The link between atypical kinase Rio1 and CK2

Our results provide another relationship between the two kinases. CK2-mediated phosphorylation of yeast Rio1 and its influence on upregulation of the enzyme were reported [44]. The authors showed that Rio1 interacts preferentially with CK2α` and phosphorylation of Rio1 promotes cell proliferation. Thus, the similar susceptibility of the two kinases to benzimidazoles creates another cross-link between the enzymes, and creates an additional condition for designing novel benzimidazole-based inhibitors of CK2.

Although it is widely reported that halogenated benzimidazoles inhibit protein kinase CK2 and induce apoptosis, the molecular mechanism by which these chemicals function in cells has not been systematically explored. Duncan and coworkers revealed that structurally related TBB, TBI, and DMAT had unique biological properties, suggesting differences in inhibitor specificity [45]. Our results suggest that proapoptotic benzimidazoles may, among many other cellular events, cause disturbances in Rio1 activity and, consequently, in ribosome biogenesis, and these events may contribute to benzimidazole-mediated programmed cell death. Koronkiewicz and coworkers showed proapoptotic activity of TIBI in the promyelocytic leukemia cell line HL-60 [39]. Taking the above into consideration, the promising findings presented here need to be extended with the use of cell lines in order to assess the influence of TIBI on endogenous Rio1.
